# Supplementary material for: The TIRS trial: Enrollment procedures and baseline characterization of a pediatric cohort to quantify the epidemiologic impact of targeted indoor residual spraying on Aedes-borne viruses in Merida, Mexico
Source: PLoS One. 2024 Sep 18;19(9):e0310480. doi: 10.1371/journal.pone.0310480 (PMC11410223; doi:10.1371/journal.pone.0310480)
Supplement: S1 Checklist — (DOCX) [file pone.0310480.s001.docx]

**WHO Trial Registration Data Set (Version 1.3.1): checklist**

*(number next to each section indicates page in protocol)*

[*https://www.who.int/ictrp/network/trds/en/*](https://www.who.int/ictrp/network/trds/en/)

| **Component** | **Present in protocol (page#)** | **If absent, description** |
| --- | --- | --- |
| 1. **Primary Registry and Trial Identifying Number** | **7** |  |
| 1. **Date of Registration in Primary Registry** | **7** |  |
| 1. **Secondary Identifying Numbers** | **7** |  |
| 1. **Source(s) of Monetary or Material Support: Primary Sponsor** | **29-30** |  |
| 1. **Contact for Public Queries** | **1** |  |
| 1. **Contact for Scientific Queries** | **1** |  |
| 1. **Public Title** | **1** |  |
| 1. **Scientific Title** | **1** |  |
| 1. **Countries of Recruitment** | **7** |  |
| 1. **Health Condition(s) or Problem(s) Studied** | **8-13** |  |
| 1. **Intervention(s)** | **8** |  |
| - 1. Intervention Description: | **13** |  |
| 1. **Key Inclusion and Exclusion Criteria** | **8-10** |  |
| 1. **Study Type**: |  |  |
| - 1. Type of study | **8** |  |
| - 1. Study design including: |  |  |
| - - 1. Method of allocation | **8** |  |
| - - 1. Masking | **N/A** | No masking |
| - - 1. Assignment (single arm, parallel, crossover or factorial) | **8** |  |
| - 1. For randomized trials: the allocation concealment mechanism and sequence generation will be documented. | **8** |  |
| 1. **Date of First Enrollment** Anticipated or actual date of enrolment of the first participant. | **8-9** |  |
| 1. **Sample Size**: |  |  |
| - 1. Number of participants that the trial plans to enroll in total. | **8-9** |  |
| - 1. Number of participants that the trial has enrolled. | **14-15** |  |
| 1. **Recruitment Status** Recruitment status of this trial: |  |  |
| - 1. Complete: participants are no longer being recruited or enrolled | **14-15** |  |
| 1. **Primary Outcome(s)** | **8** |  |
| 1. **Key Secondary Outcomes** | **8** |  |
| 1. **Ethics Review**: |  |  |
| - 1. Status: Approved | **7** |  |
| - 1. Date of approval | **7** |  |
| - 1. Name and contact details of Ethics committee(s) | **7** |  |
| 1. **Completion date** Date of study completion: The date on which the final data for a clinical study were collected (commonly referred to as, "last subject, last visit"). | **N/A** | Trial is ongoing |
| 1. **Summary Results**: |  |  |
| - 1. Date of posting of results summaries | **N/A** | Not yet available |
| - 1. Date of the first journal publication of results | **N/A** | Not yet published |
| - 1. URL hyperlink(s) related to results and publications | **N/A** | Not yet published |
| - 1. Baseline Characteristics: | **16** | Table 1 |
| - 1. Participant flow: | **14-15** |  |
| - 1. Adverse events: | **N/A** | None observed |
| - 1. Outcome measures: | **22-26** |  |
| - 1. URL link to protocol file(s) with version and date | **9** |  |
| - 1. Brief summary |  |  |
| 1. **IPD sharing statement** |  |  |
| - 1. Plan to share IPD (Yes, No) | **14** |  |
| - 1. Plan description | **14** |  |
