# Supplementary figures and images for: The TIRS trial: Enrollment procedures and baseline characterization of a pediatric cohort to quantify the epidemiologic impact of targeted indoor residual spraying on Aedes-borne viruses in Merida, Mexico
Source: PLoS One. 2024 Sep 18;19(9):e0310480. doi: 10.1371/journal.pone.0310480 (PMC11410223; doi:10.1371/journal.pone.0310480)

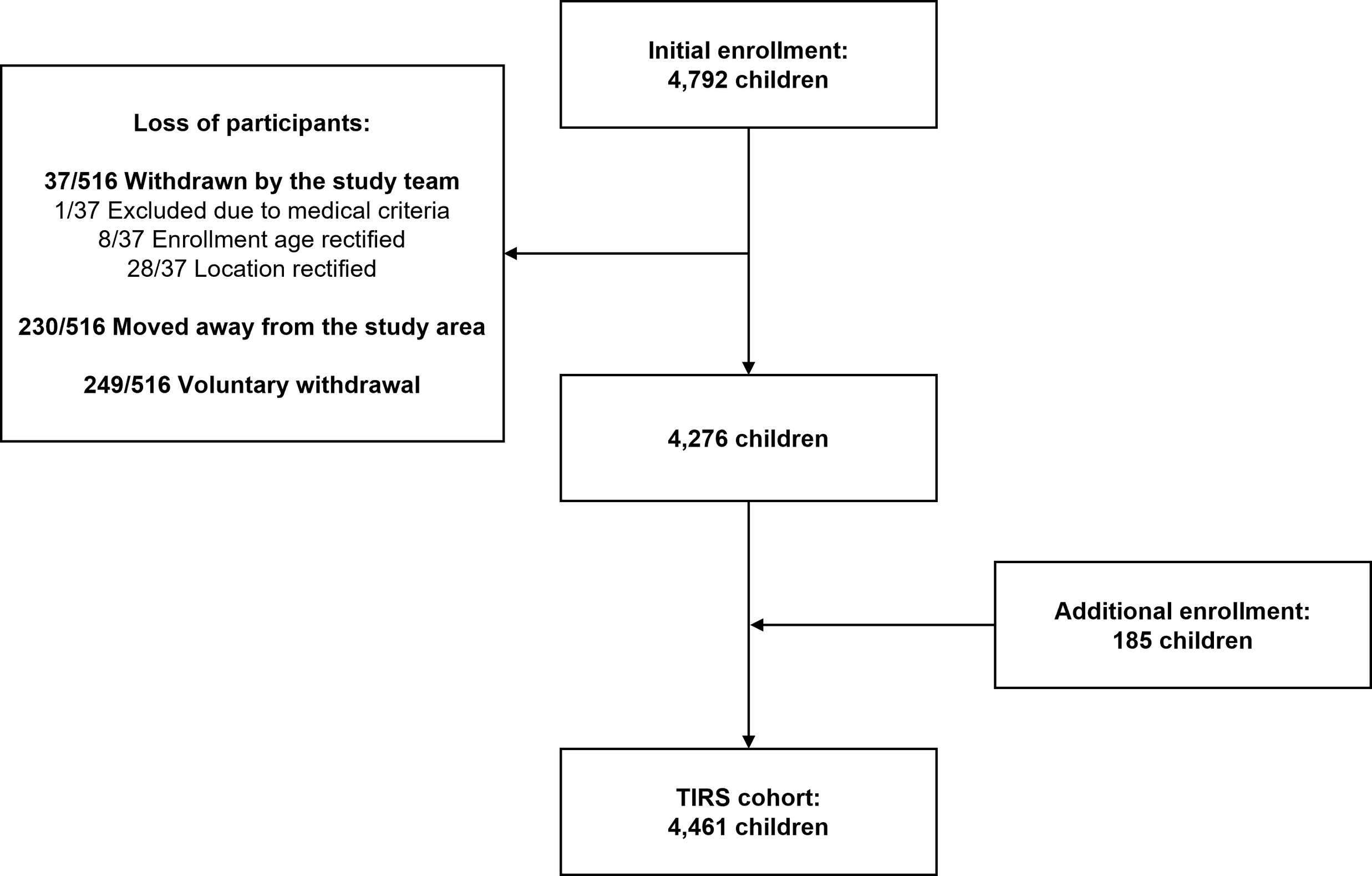

Supplement: S1 Fig — (TIF) [file pone.0310480.s002.tif]

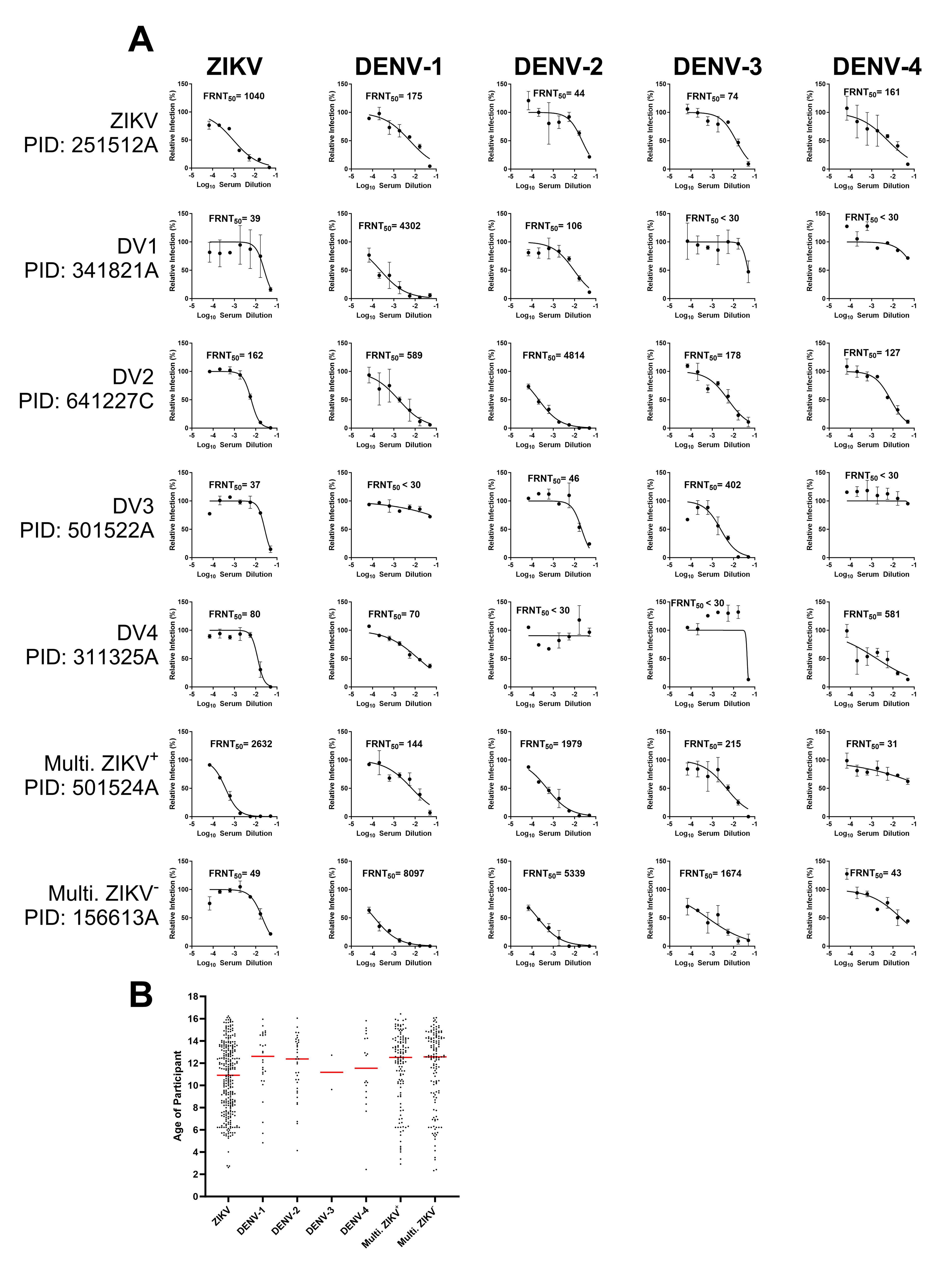

Supplement: S2 Fig — (A) FRNT curves and calculated FRNT50 values for representatives of the indicated monotypic or multitypic exposure groups (left) against the virus indicated (top) for individual participants (participant ID [PID] indicated on left). (B) Ages of participants at time of sampling (median in red). (TIF) [file pone.0310480.s003.tif]
