# Supplementary material for: The TIRS trial: Enrollment procedures and baseline characterization of a pediatric cohort to quantify the epidemiologic impact of targeted indoor residual spraying on Aedes-borne viruses in Merida, Mexico
Source: PLoS One. 2024 Sep 18;19(9):e0310480. doi: 10.1371/journal.pone.0310480 (PMC11410223; doi:10.1371/journal.pone.0310480)
Supplement: Attachment — Submitted filename: 091523 PNTD Rebuttal.docx [file pone.0310480.s004.docx]

15 September 2023

Dear Dr. Abd-Alla and Dr. Samy,

Thank you for providing the reviewers’ comments for our manuscript describing the baseline results of our field trial. We are excited that the study was well received and grateful for the assistance provided by the reviewers. I have responded to reviewer comments in line in this document (in red text) with line numbers that correspond with the track changed word document. Any changes that are not called out specifically in the response below are a result of thorough copyediting to improve readability but do not affect the interpretation of the study. Thank you for consideration of our manuscript. I hope that we have addressed the issues identified by the reviewers. Please let me know if there is anything else I can do.

Sincerely,

Gonzalo Vazquez-Prokopec

Department of Environmental Sciences

Emory University

400 Dowman Drive

Atlanta, GA 30030

(404) 727-4217

**Response to Reviewer’s Comments**

Key Review Criteria Required for Acceptance?
As you describe the new analyses required for acceptance, please consider the following:

**Methods**
-Are the objectives of the study clearly articulated with a clear testable hypothesis stated?
-Is the study design appropriate to address the stated objectives?
-Is the population clearly described and appropriate for the hypothesis being tested?
-Is the sample size sufficient to ensure adequate power to address the hypothesis being tested?
-Were correct statistical analysis used to support conclusions?
-Are there concerns about ethical or regulatory requirements being met?

Reviewer #1: The objectives of the study are clearly articulated, and this publication’s principal objective appears to demonstrate that Treatment and Control areas for a large TIRS CRCT are well balanced at baseline.  The manuscript achieves this goal and provides additional insight about a trial that had to adjust in “real time” to the COVID-19 pandemic.  The study design is very rigorous and has been published previously (reference 22).  The statistical analysis also appears rigorous and appropriate.  Ethical requirements have been met.
The authors thank the reviewer for the kind words.

Minor criticisms include:
The effect size that the study is being powered for is not clearly stated, probably just an omission (see specific comments below) but should be included.
We included a statement in the methods to address this (lines 187-188). As indicated in Manrique-Saide etal (2020) we powered the study to detect a TIRS efficacy of 70%, which means the trial will need to collect a minimum of 92 events. The more events above 92, the higher the effect size that will be ascertained.

Many of the methodological strengths of the trial are mentioned in the last paragraph of the introductions, but details are not included in this manuscript.  I would encourage the authors to explain why the covariate-constrained randomization is advantageous beyond reducing the number of clusters required, which is on the high side compared to other CRCT trials using epidemiological endpoints for dengue.  I see this more as an opportunity than a requirement.

We thank the reviewer for pointing out this opportunity. The justification and advantages of the covariate-constrained randomization have been expanded upon in both the introduction (lines 126-141) and conclusions (lines 573-578).

Reviewer #2: In the abstract, both tin line 47 and line 52, the number of enrolled children is mentioned. I suggest grouping this information so as to not have to repeat it.

Lines 47-48 and 53-55 in the abstract have been edited to remove this redundancy.

In line 56, the authors group together the prevalence of children with DENV or ZIKV, although this is explained in the body of the manuscript, given that flavivirus were grouped together due to cross reactivity, if someone only reads the abstract, it's confusing. Please state in a more clear manner.

The text has been changed from “either DENV or ZIKV” to “one or more flaviviruses” to avoid confusion (line 58).

Line 61: Either place a semicolon after compositions or , i.e. Ae. aegypti infestation and susceptibility to insecticides. Author summary –

Lines 62-65 in the abstract were altered to make this more readable.

Line 66: Shouldn't all the viruses be in capital letters? Why Zika en capital letters but not Dengue and Chik?
Introduction: Line 82: again capitalization of virus names.

While we agree this looks awkward, these are the capitalization conventions for these viruses.

Line 133 - here it stated that the TIRS trial is ongoing, I suggest stating start and end date somewhere.

The start and end dates have been added to line 143.

Methods: The objectives, study design, sample size, statistical analysis and regulatory requirements are met, but I have several suggestions/questions.
Line 148 - Given the seasonal pattern of infection in the study site, it would be good to mention that the period to measure seroprevalence is during the inter-epidemic period, explain the timing of the interventions.

The transmission period has been made specific (line 159) and the time of seroprevalence survey has also been included (lines 230-231).

Line 152-153 - person-years is used for DENV but not for CHIK or ZIKV and also standardize how you mention de viruses, here you state DENV, chikungunya and Zika.

We altered all numbers to mention person-years to ensure consistency (line 161-163)

Line 153 - Aedes aegypti infests houses or Aedes aegypti mosquitoes infest houses...

This line now reads “Aedes aegypti mosquitoes infest houses…” (line 164).

Study Design Line 163 -  will be, it's not clear given the different verbal tenses used, if the trial started or not, or what part has started and what part is still yet to begin.

Lines 173-183 were written more clearly to indicate that the trial has started and is ongoing.

Lines 169 - 178: The methodological part is very long and confusing. Given you have already published the protocol for this study (ref 22) and you have already stated there all the methodology and sample sizes calculations, in this manuscript, I suggest concentrating on the baseline data which is what you actually report herein.

The “study design” section was shortened greatly to emphasize relevant information (lines 173-195). We also removed a great deal of methodology to emphasize only methods relevant to this study (e.g. lines 216-221, 236-245, etc)

Participant Enrollment and Impact of COVID-19 - this subpart of the method sections could also be shortened.

These methods have also been shortened to focus only on this study and to enhance clarity.

Line 212 - I think you forgot to put the year here? Was it 2020?

Yes, the year was 2020 and has been corrected in the text (line 230).

Baseline sources of information - This is the actual methodology for the data you are reporting herein. The previous paragraphs could all be shortened.

We have included extensive revisions of the methods section and hope it is more clear now.

Line 256 - Are there any previous studies with these commercially available ELISAs that have validated their performance?

References (#s 30, 36, 44, and 45) for previous studies using these kits have been included in line 285.

Line 260 - These were also IgG ELISAs? Please make sure the laboratory methodology is clear and concise. Is there a copyright or more information on the Euroimmun tests? Remember that when mentioning commercial tests you include the copyright information as well so that a fellow researcher can find exactly the same test used.

We have rewritten this section to include which type of ELISA was performed as well as the company producing the kits and the catalog numbers for each kit (line 273-286).

Reviewer #3: The objective, study design, population, sample size, analysis everything has been described nicely. But the problem is that the study has not been done and results of study are not given. Only methods of study have been described which should be part of main research paper. hence it can't be called a research or study. It is only a part of the study, which has to be conducted. That is also reflected in the title.
We appreciate this feedback. We have greatly reduced the length of our methods section to reflect only what is presented in this manuscript (e.g. lines 216-221, 236-245, etc) and refer readers to the previously published protocol that has greater methodologic information (ref 22). We agree and understand that the pivotal data on TIRS efficacy will only come when the trial is complete. However, we believe the current study has substantial value *per se* in reporting important epidemiologic and entomologic data for this arbovirus-endemic region.

**Results**
-Does the analysis presented match the analysis plan?
-Are the results clearly and completely presented?
-Are the figures (Tables, Images) of sufficient quality for clarity?

Reviewer #1: The results are clearly presented, figure quality is high, but I would consider including a bit more information in the figure and table legends, in particular reminding the reader that the analysis is blinded, and Arms A and B distinguish between treatment and control.  Again, a minor issue.

We have made changes in the legends for all figures and tables to make it more clear what the Arms represent.

Reviewer #2: Participant Enrollment - A flowchart of enrollment would greatly clarify the cascade and reasons for loss of participants and addition of new ones.

A flowchart describing loss to followup has been included as Fig S1.

Table 3, line 418 - Table 3 states an age range of 13 to 16 and not 15 as in the text. The protocol specifies enrollment up to 15 years of age. Please clarify.

These discrepancies are due to participants who turned 16 between enrollment and baseline sampling. We have changed the text to reflect that the age is at time of baseline sampling. We would prefer to keep it this way as the cohort will age over time and comparisons at the end of the trial will be easier with the data presented this way. We have altered the legend for Table 3 and the text in the results (line 452-454) to reflect this.

Lines 418 to 422 - Report in the same format as flavivirus, with the number of positive and the percentage and then specifying the age of the younger and older cohort. Please standardize for clarity.

This text has been changed as suggested (line 456-459)

Line 437 - When you say serotypes it sounds as if you refer only to Dengue serotypes, for clarity, I would suggest stating participants with antibodies to more than one flavivirus or to more than one DENV serotype were considered multitypic.

The text in lines 497-499 has been changed to make this more clear

Line 437 - mutitypic should be multitypic

This typo has been corrected (line 503)

Line 441 to 446 - This sounds more like part of the discussion, not results.

The speculative text has been removed (lines 506-508). The age stratified ZIKV-specific results are still presented in lines 508-512.

Figure 3. D should say multytipic

This typo was fixed in Fig 3D

Figure 4 - Given that the same legend is suitable for all the years, I would suggest including it only once or even in the written legend you could specify the colors for each arm. It's confusing to see the legend for every year because readers might think they are representing different things for each year.

The legend has been included only once in the revised Fig 4.

Reviewer #3: See above

**Conclusions**
-Are the conclusions supported by the data presented?
-Are the limitations of analysis clearly described?
-Do the authors discuss how these data can be helpful to advance our understanding of the topic under study?
-Is public health relevance addressed?

Reviewer #1: Conclusions are supported by the data presented.
The authors discuss the problems with loss to follow up, which they attribute to COVID-19, although I would argue these rates are consistent with those found elsewhere.
Where this manuscript may fall a little bit short is how it is advancing our understanding of ABV transmission or TIRS, it will provide the authors the opportunity to cite this manuscript in future publications to state that covariates were balanced at baseline, but again I think they could better make the argument that the balance is a result of their careful study design.

We thank the reviewer for their feedback regarding the scope of these analyses. We make no claims that this manuscript is a thorough investigation of ABV transmission in an endemic area or an analysis of the efficacy of TIRS. Future analyses that use these baseline datasets will provide much more insight on the longitudinal changes within such a population.

Another observation, was that the Aedes aegypti data (adults), showed very little heterogeneity, which I felt was somewhat unique.  It would be useful for the authors to state what the expected impact of TIRS on those densities needs to be.

Thank you for this observation. We conducted the adult mosquito baseline in the dry season, prior to the seasonal increase in mosquito numbers. One of the reasons why the mosquito numbers look invariable is because populations were low during the dry season (when population size is small). The goal with this figure was to show the balance between arms, rather than any information related to the possible impact of TIRS. We generally find increases in density of up to 10x compared to the dry season, so the expected impact will be measured by monthly entomological surveys post-TIRS. Similarly, combining adult mosquitoes and ovitrap data provided information to help guide the readers about the balance of the study arms with regards to entomology. This is a numerical estimate that confirms that the covariate constrained randomization minimized any potential bias that may have resulted in arms having different *Ae. aegypti* density.

Reviewer #2: Lines 533 to 538 - I suggest being more specific, given that not all countries and not all cities had the same response to the pandemic or implemented non-pharmacological interventions at the same time. Which was the timeline for Merida specifically with respect to lockdown, social-distancing and back to school activities. In the Americas the pandemic occurred during most of 2020 but many countries were no longer in lockdown by 2021. It's important to characterize this specifically for your study area. This applies to the methodology section as well.

This is a valid point. Reference #41, co-authored by our team, provides a detail of the type of COVID-19 interventions and their timing in Merida. We now include some of that detail, related to the timing of interventions during our trial. Just for information, Merida kept a stringent COVID-19 policy for a longer period than the US, primarily due to initial low vaccine availability. So, NPIs were continued throughout most of 2021.

Lines 541 to 543 - I am not sure this is relevant to the study.

This sentence has been removed (line 587-589)

Lines 550 to 555 - Although interesting, I would stick to the actual results of the study.

We respect the reviewer’s criticism and understand the reasoning, but we believe that the techniques used by the TIRS trial to carry out a multinational field study during the COVID-19 pandemic may be useful for other trials in the future. Thus, we have decided to include these details.

Line 572 - Begins sentence should be Aedes...

This has been corrected (line 618).

Figure S1 - This a very extensive and busy figure which is hard to visualize. Given that the study protocol has been already published, I don´t think it add much to the manuscript in the current form.
Figure S2 - I understand this is a form that will be used to monitor the phases of the study trial, here you are informing the baseline data, I suggest removing it as it does not add to the manuscript.
We appreciate the feedback and agree with your concerns. Because of this, we have removed Figs S1 and S2.

Reviewer #3: See above.


**Editorial and Data Presentation Modifications?**

Reviewer #1: This manuscript would benefit from another round of copy editing.

We hope to have fixed any typos or unclear statements with a thorough editing of the text and figures.

Reviewer #2: The authors state in their data availability statement that "The data that support the findings of this study are publicly available from Mendeley data (doi: 10.17632/jtm6j2rmx9.1)". But the link does not work.

The data are uploaded to Mendeley but are under embargo until the paper is published. At the time of acceptance/publication, the embargo will be lifted and the datasets will be fully available.

Reviewer #3: See above.


**Summary and General Comments**
Use  this section to provide overall comments, discuss strengths/weaknesses of the study, novelty, significance, general execution and scholarship. You may also include additional comments for the author, including concerns about dual publication, research ethics, or publication ethics. If requesting major revision, please articulate the new experiments that are needed.

Reviewer #1: General Comments
See above sections
Specific Comments

Lines 167-169:  Sentence awkward and unclear.  This sounds like two endpoints to me. Seroconversion, which does require a definition or explanation.  For example, do you mean seroconversion to specific viruses including each DENV serotype.  Exposure implies seroprevalence also virus/serotype specific, which is not what you mean in reference 22.  Suggest changing to:
“The secondary epidemiological endpoints include virus and for DENV serotype specific seroconversion rates from annual blood draws from the cohort participants (2-15 years of age).”

We have changed the text of this section of the methods to be more clear about our trial endpoints (lines 177-185). We have also significantly edited the methods to be more concise as suggested by multiple reviewers.

Line 174: state the effect size the trial is powered for.

This has been added to the methods (lines 187-188).

Line 186: Do you mean N95 masks?

Yes, this has been corrected (line 204)

Line 212 (what year)? Indeed this need to be filled in, but assuming it is 2020?
Yes, 2020. This text has been corrected (line 230)

Line 354-356: Values presented in the text are not the same as Table 1, and please remind us what Arm A and Arm B represent?  I had to go back and look.

The text has been corrected (line 386-388) and the legend for Table 1 has been changed to reflect that Arm A and Arm B represent the blinded control and interventions arms.

Reviewer #2: This study by Earnest and collaborators describes the enrollment procedures and baseline characterization of cohort of children between the age of 2 and 15 from the city of Merida in Mexico were an ongoing Targeted Indoor Residual Spraying (TIRS) trial is being conducted. The protocol for the study itself has been previously published by the authors (Manrique-Saide et al., 2020). Given that this protocol has been previously published and this specific manuscript details the baseline data collected and analyzed required to be able to measure the impact of the intervention planned in the TIRS trial, I suggest major revision to focus specifically on that. This will greatly improve the clarity and help better highlight the excellent results obtained.

We thank you for your feedback and hope that our revisions have enhanced the clarity of the manuscript

Reviewer #3: See above.
